# Sites of Distant Metastases and Cancer-Specific Survival in Intraductal Papillary Mucinous Neoplasm With Associated Invasive Carcinoma: A Study of 1,178 Patients

**DOI:** 10.3389/fonc.2021.681961

**Published:** 2021-06-03

**Authors:** Xiaoyi Huang, Siting You, Guiling Ding, Xingchen Liu, Jin Wang, Yisha Gao, Jianming Zheng

**Affiliations:** ^1^ Department of Pathology, Changhai Hospital, Second Military Medical University, Shanghai, China; ^2^ Central Laboratory, Changhai Hospital, Second Military Medical University, Shanghai, China

**Keywords:** IPMN, metastases, survival, SEER, chemotherapy, tumor location, nomogram

## Abstract

**Background:**

To explore the impact of distant metastases on cancer-specific survival in patients with intraductal papillary mucinous neoplasm (IPMN) with associated invasive carcinoma and identify the risk factor of distant metastases in IPMN with associated invasive carcinoma.

**Methods:**

Patients with IPMN with associated invasive carcinoma between 2010 and 2015 were retrospectively selected from the Surveillance, Epidemiology, and End Results (SEER) database. The survival analyses were assessed by Kaplan-Meier analyses and log-rank test. The impact of distant metastases was evaluated by Cox regression model and the risk factors of distant metastases were identified by logistic regression analyses, respectively.

**Results:**

The median cancer-specific survival time of patients with no metastases, isolated liver, isolated lung, and multiple site metastases were 19 months, 4 months, 7 months, and 3 months, respectively. In patients with isolated liver metastases, multivariate analysis after adjustment indicated that chemotherapy (Hazard Ratio [HR]=0.351, 95% confidence interval [CI]=0.256-0.481, P<0.001) was a protective prognostic factor for cancer-specific survival (CSS) in patients with isolated liver metastases. In isolated lung metastases subgroup, old age (HR=1.715, 95% CI=1.037-2.838, P=0.036) and chemotherapy (HR=0.242, 95% CI=0.134-0.435, P<0.001) were related to CSS in multivariable Cox regression analysis(P<0.05). Tumor located in the pancreatic body/tail (HR=2.239, 95% CI=1.140-4.400, P=0.019) and chemotherapy (HR=0.191, 95% CI=0.108-0.340, P<0.001) were independent prognostic factors for CSS in patients with multiple metastases. Finally, a nomogram was constructed for cancer-specific survival and the predicted C-index was 0.780 (95% CI=0.762-0.798).

**Conclusion:**

The liver is the most common site of distant metastases in IPMN with associated invasive carcinoma. Tumor located in the pancreatic body/tail and chemotherapy are independent prognostic factors for CSS in patients with multiple metastases. Further, tumor located in body/tail is identified as a risk factor of distant metastases.

## Introduction

Intraductal papillary mucinous neoplasms (IPMN) is defined as a tumor arising from the ductal epithelium, characterized by mucin production, cystic dilation of the pancreatic duct, and intraductal papillary growth (1). IPMNs exhibit a spectrum of neoplastic transformation ranging from hyperplasia to invasive carcinoma (2). Surgery is recommended for patients with high malignant risk. Related invasive carcinoma can be detected in 40%-60% of IPMN resected lesions (3). IPMN with associated invasive carcinoma has an unfavorable prognosis with a 5-year overall survival of 20%-40%, while that of noninvasive IPMNs is over 90% (4, 5).

Distant metastasis is a characteristic of malignant tumors. According to previous studies, lymph node metastases occurred in 5–54% of patients with IPMN with associated invasive carcinoma (6, 7). Unfavorable prognoses have been reported for IPMN with associated invasive carcinoma with lymph node metastases (8). However, previous studies reported that the rate of lymph node metastases in IPMN with associated invasive carcinoma is lower than that in pancreatic ductal adenocarcinomas (7, 9). As for distant organ metastases, there are very few studies on IPMN with associated invasive carcinoma with distant metastases. As IPMNs are considered “pre-invasive lesions” of pancreatic ductal adenocarcinoma, whether the pattern of distant metastases in IPMN with associated invasive carcinoma the same as in pancreatic ductal adenocarcinoma is unclear yet. Besides, the accurate ratio of distant metastases and the effect on survival in IPMN with associated invasive carcinoma have not yet been reported. Therefore, this retrospective study aimed to evaluate the prognostic value of the distant metastases on IPMN with associated invasive carcinoma and to analyze the outcomes of patients with IPMN with associated invasive carcinoma based on the Surveillance, Epidemiology, and End Results (SEER) database.

## Methods and Materials

**Figure 1:**
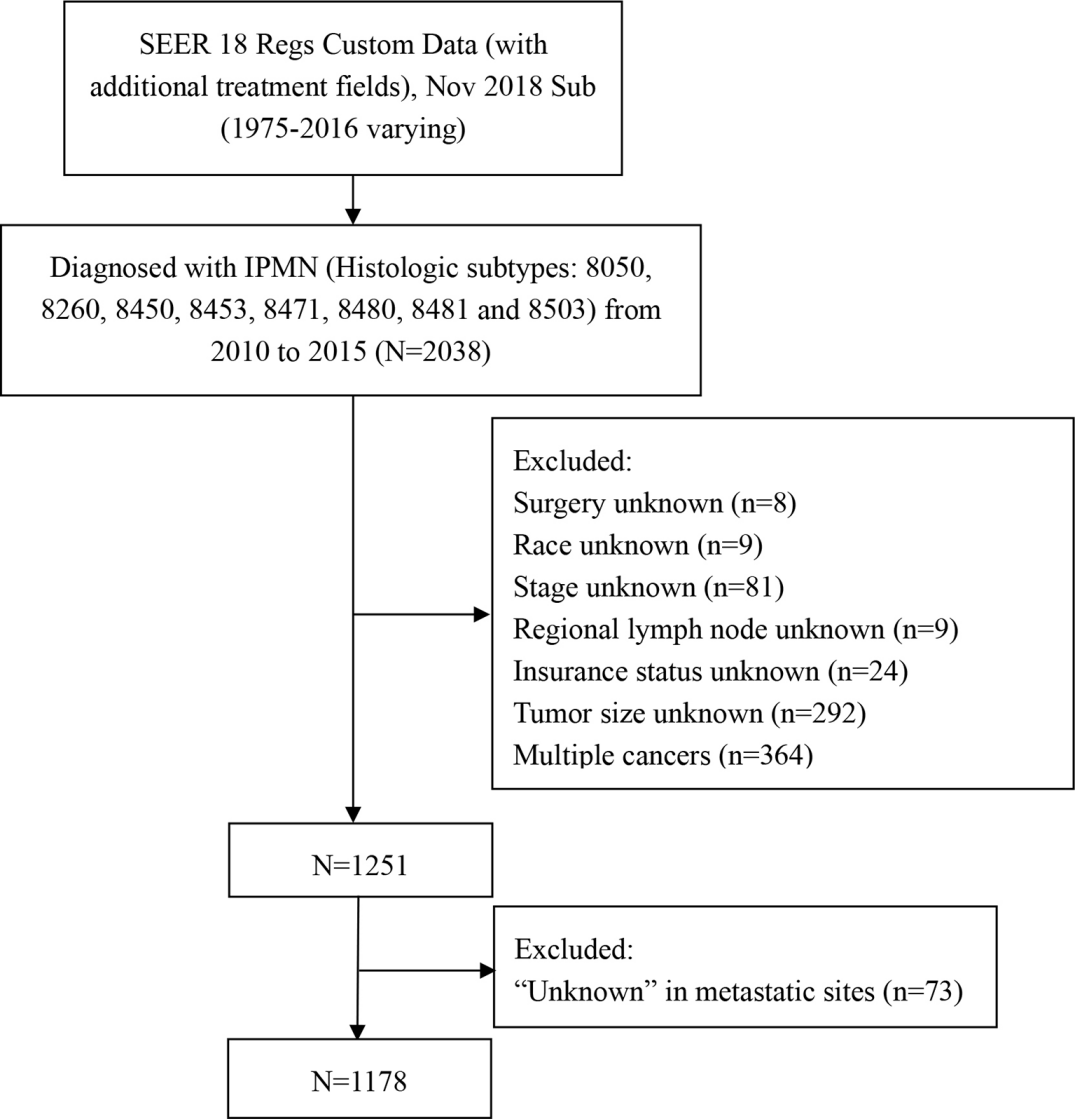
The flow chart of eligible patients’ selection in this study.

A retrospective cohort study was performed by extracting the data from the SEER database. The SEER database is free, and informed consent was waived because the SEER data are anonymous, and the study complied with the cancer is a reportable disease in every state of the USA. The incidence-SEER 18 Regs Custom Data (with additional treatment fields), Nov 2018 Sub (1975-2016 varying) was employed as the data source. The 5-year relative survival rate was extracted from SEER 18 Regs Custom Data (with additional treatment fields) from 2000 to 2016. Because the SEER database is reasonable for the classification of invasive carcinomas, only patients who had a histologic diagnosis as IPMN with associated invasive carcinoma of the pancreas were included. We selected cases through the “SEER Site Recode” using the term “pancreas” and identified along with the label “malignant” using the variable “Histologic Type ICD-O-3” with the following codes: 8050, 8260, 8450, 8453, 8471, 8480, 8481 and 8503 (10). Patients were excluded if they were diagnosed at autopsy or *via* death certificate, without detailed information about clinical characteristics. The flow chart of the selection for the study group was summarized in **Figure 1**. Demographic and clinical data were extracted for every patient, including age at diagnosis, gender, race, primary site, histologic grade, surgery, radiotherapy, chemotherapy, marital status, distant organ metastases, tumor size, and survival duration. Radiotherapy and chemotherapy were classified as “yes” or “no/unknown” in the database. The sites of distant metastases were classified as bone, brain, liver, and lung. Because the SEER database only included distant metastases information from 2010, so we identified cases in the year of diagnosis from 2010 to 2015. We assessed the relationship between site-specific metastatic sites and survival.

### Statistical Analysis

Statistical analyses were performed using SPSS (version 22.0 IBM Corporation, Armonk, NY). Categorical variables were reported as counts. Independent predictors for cancer-specific survival were identified by univariate and multivariable Cox proportional hazard models. Kaplan-Meier analysis and log-rank test were used to compare survival duration between different subgroups. Distant metastasis-associated factors were identified by logistic regression models. A two-sided P value <0.05 was considered statistically significant in all analyses. The validated variables were incorporated into the nomogram to predict the probability of 1‐, 3‐, and 5‐year CSS rates for patients with IPMN with associated invasive carcinoma using the rms package in R software (http://www.r-project.org/). The discrimination and calibration of the nomogram were evaluated using the concordance index (C-index) and calibration curve.

## Results

### Characteristics of Patients With IPMN With Associated Invasive Carcinoma

A total of 1178 patients with IPMN with associated invasive carcinoma that met our criteria were included in the analysis. Among them, 10 patients (0.85%) with isolated bone metastases, 245 (20.80%) with isolated liver metastases, 94 (8.0%) with isolated lung metastases, and 107 (9.1%) with multiple metastases. The distant metastases rate was 38.7% in patients with IPMN with associated invasive carcinoma. Patients were diagnosed at the age of more than 65 (55.8%), male (50.4%), white (78.8%), and married (55.3%). Regarding the primary site, 547 (46.4%) patients with tumor located in the pancreatic head, and 34.5% in the pancreatic body/tail. As for therapeutic options, most patients did not undergo surgery (68.7%) or radiotherapy (83.4%), while 686 (58.2%) received chemotherapy. The demographic and clinicopathological characteristics of patients with IPMN with associated invasive carcinoma are summarized in **Table 1**.

**Table 1:** Clinical characteristics of patients with IPMN with associated invasive carcinoma.

Variables	Total	No metastasis	Metastatic site			
			Bone	Liver	Lung	Multiple site
Age	1178	722	10	245	94	107
≤65	521 (44.2%)	328 (45.4%)	3 (30%)	114 (46.5%)	37 (39.4%)	40 (37.4%)
>65	657 (55.8%)	394 (54.6%)	7 (70%)	131 (53.5%)	58 (61.7%)	67 (62.6%)
Gender						
Male	594 (50.4%)	370 (51.2%)	5 (50%)	128 (52.2%)	43 (45.7%)	48 (44.9%)
Female	584 (49.6%)	352 (48.8%)	5 (50%)	117 (47.8%)	51 (54.3%)	59 (55.1%)
Race						
White	928 (78.8%)	555 (76.9%)	10 (100%)	198 (80.8%)	79 (84.0%)	86 (80.4%)
Black	121 (10.3%)	79 (10.9%)	0	23 (9.39%)	8 (8.5%)	11 (10.3%)
Other	129 (10.9%)	88 (12.2%)	0	24 (9.8%)	7 (7.4%)	10 (9.3%)
Site						
Head	547 (46.4%)	415 (57.5%)	5 (50%)	72 (29.4%)	34 (36.2%)	21 (19.6%)
Body/tail	406 (34.5%)	185 (25.6%)	1 (10%)	118 (48.2%)	45 (47.9%)	57 (53.3%)
Other	225 (19.1%)	122 (16.9%)	4 (40%)	55 (22.4%)	15 (16.0%)	29 (27.1%)
Grade						
I	139 (11.8%)	119 (16.5%)	1 (10%)	7 (2.9%)	6 (6.4%)	6 (5.6%)
II	231 (19.6%)	184 (25.5%)	2 (20%)	28 (11.4%)	8 (8.5%)	9 (8.4%)
III	124 (10.5%)	90 (12.5%)	0	20 (8.2%)	5 (5.3%)	9 (8.4%)
IV	6 (0.5%)	5 (6.9%)	0	1 (0.4%)	0	0
Unknown	678 (57.5%)	324 (44.9%)	7 (70%)	189 (77.1%)	75 (79.8%)	83 (77.6%)
Histologic type						
Mucinous adenocarcinoma	786 (66.7%)	482 (66.8%)	9 (90%)	156 (63.7%)	69 (73.4%)	70 (65.4%)
Mucin-producing adenocarcinoma	220 (18.7%)	81 (11.2%)	1 (10%)	83 (33.9%)	20 (21.3%)	35 (32.7%)
Intraductal papillary-mucinous carcinoma	126 (10.7%)	123 (17.0%)	0	2 (0.8%)	1 (1.1%)	0
Others*	46 (3.9%)	36 (5.0%)	0	4 (1.6%)	4 (4.3%)	2 (1.9%)
Surgery						
Yes	369 (31.3%)	360 (49.9%)	1 (10%)	239 (97.6%)	1 (1.1%)	1 (0.9%)
No	809 (68.7%)	362 (50.1%)	9 (90%)	6 (2.4%)	93 (98.9%)	106 (99.1%)
Radiotherapy						
Yes	195 (16.6%)	155 (21.5%)	5 (50%)	236 (96.3%)	8 (8.5%)	18 (14.0%)
No	983 (83.4%)	567 (78.5%)	5 (50%)	9 (3.7%)	86 (91.5%)	89 (83.2%)
Chemotherapy						
Yes	686 (58.2%)	426 (59.0%)	5 (50%)	104 (42.4%)	61 (64.9%)	53 (49.5%)
No	492 (41.8%)	296 (41.0%)	5 (50%)	141 (57.6%)	33 (35.1%)	54 (50.5%)
Marital status						
Married	652 (55.3%)	420 (58.2%)	5 (50%)	117 (47.8%)	53 (56.4%)	57 (53.3%)
Unmarried	468 (39.7%)	277 (38.4%)	5 (50%)	110 (44.9%)	30 (31.9%)	46 (43.0%)
Unknown	58 (4.9%)	25 (3.4%)	0	18 (7.3%)	11 (11.7%)	4 (3.7%)

*Including papillary carcinoma, papillary adenocarcinoma, papillocystic adenocarcinoma, papillary mucinous cystadenocarcinoma, and noninfiltrating intraductal papillary adenocarcinoma.

### Survival Analysis

**Figure 2:**
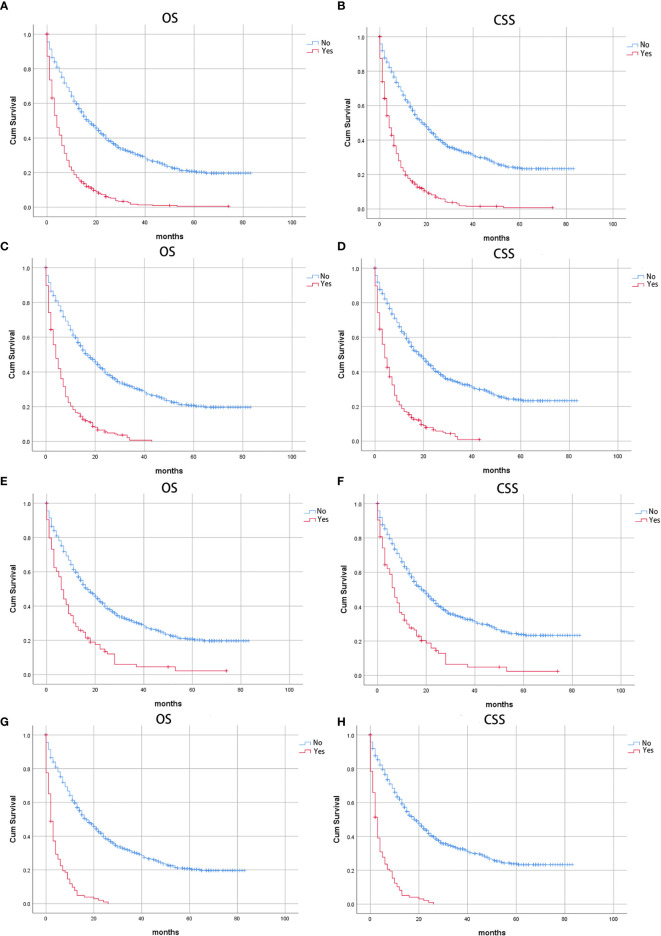
Kaplan-Meier curves of patients with IPMN with associated invasive carcinoma: Stratified by distant metastases **(A)** Overall survival (OS) and **(B)** Cancer-specific survival (CSS); stratified by liver metastases, **(C)** Overall survival (OS) and **(D)** Cancer-specific survival (CSS); stratified by lung metastases, **(E)** Overall survival (OS) and **(F)** Cancer-specific survival (CSS); stratified by multiple metastases, **(G)** Overall survival (OS); **(H)** Cancer-specific survival (CSS).

The CSS of patients with single or multiple distant metastases involvements were compared according to the distant site. In patients with no distant metastases, liver, lung, and multiple metastases, the median CSS was 19 months, 4 months, 7 months, and 3 months, respectively. And the median OS in patients with no distant metastases, liver, lung, and multiple metastases was 17 months, 4 months, 4 months, and 2 months, respectively. The Kaplan-Meier curves were shown in **Figure 2**. Further, the 5-year relative survival rate in IPMN with associated invasive carcinoma with distant metastases was calculated by SEER*Stat and the results showed that the 5-year relative survival was 1.7%. The 5-year relative survival rate in all stages of IPMN with associated invasive carcinoma was 9.7%. Further, to identify the predictors associated with survival of patients with IPMN with associated invasive carcinoma, the Cox regression was employed. In the no metastasis subgroup, the univariable Cox regression analysis indicated that age, gender, primary site, histologic grade, histologic subtypes, surgery, radiotherapy, and marital status were correlated with CSS (P<0.05). The multivariate analysis after adjustment revealed that tumor located in the pancreatic body/tail (HR=1.337, 95% CI=1.077-1.659, P=0.008), histologic subtypes (mucinous adenocarcinoma: HR=0.698, 95% CI=0.534-0.913, P=0.009; intraductal papillary-mucinous carcinoma: HR=0.350, 95%CI=0.228-0.538, P<0.001), surgery (HR=0.218, 95% CI=0.171-0.278, P<0.001), radiotherapy (HR=0.663, 95% CI=0.523-0.841, P=0.001), and chemotherapy (HR=0.770, 95% CI=0.620-0.956, P=0.018) were independently prognostic factor for CSS in patients with IPMN with associated invasive carcinoma. In patients with isolated liver metastases, the univariable Cox regression analysis indicated that surgery and chemotherapy were associated with CSS (P<0.05). The multivariate analysis after adjustment indicated that chemotherapy (HR=0.351, 95% CI=0.256-0.481, P<0.001) was a protective prognostic factor for CSS in patients with isolated liver metastases. In isolated lung metastases subgroup, old age (HR=1.715, 95% CI=1.037-2.838, P=0.036) and chemotherapy (HR=0.242, 95% CI=0.134-0.435, P<0.001) were related to CSS in multivariable Cox regression analysis(P<0.05). As for the multiple metastases subgroup, univariable Cox regression analysis showed that age, chemotherapy, and marital status were associated with CSS (P<0.05). In multivariable analysis, tumor located in the pancreatic body/tail (HR=2.239, 95% CI=1.140-4.400, P=0.019) and chemotherapy (HR=0.191, 95% CI=0.108-0.340, P<0.001) were independent prognostic factors for CSS in patients with IPMN with associated invasive carcinoma. The details of Cox regression analyses were summarized in **Tables 2**, **3**.

**Table 2:** Univariable Cox regression analysis of cancer-specific survival in patient with IPMN with associated invasive carcinoma.

Variables	No metastasis		liver		lung		Multiple metastases	
	HR (95% CI)	P	HR (95% CI)	P	HR (95% CI)	P	HR (95% CI)	P
Age								
≤65	1		1		1		1	
>65	1.245 (1.038-1.494)	0.018	1.084 (0.834-1.409)	0.545	1.892 (1.180-3.033)	0.008	1.526 (1.008-2.308)	0.046
Gender								
Male	1		1		1		1	
Female	1.315 (1.096-1.577)	0.003	1.063 (0.818-1.382)	0.646	0.839 (0.544-1.294)	0.426	1.221 (0.820-1.817)	0.326
Race								
White	1		1		1		1	
Black	1.014 (0.758-1.355)	0.928	0.882 (0.561-1.386)	0.586	0.588 (0.236-1.466)	0.254	0.698 (0.348-1.399)	0.310
Other	0.658 (0.481-0.899)	0.009	0.659 (0.415-1.047)	0.078	0.774 (0.335-1.790)	0.550	1.136 (0.586-2.204)	0.706
Primary site								
Head	1		1		1		1	
Body/tail	1.515 (1.229-1.867)	<0.001	1.009 (0.743-1.371)	0.953	1.034 (0.641-1.668)	0.890	1.725 (0.989-3.007)	0.055
Other	1.119 (0.866-1.445)	0.389	1.179 (0.819-1.698)	0.375	1.528 (0.804-2.903)	0.196	1.962 (1.068-3.602)	0.030
Histologic grade								
I	1		1		1		1	
II	1.676 (1.207-2.327)	0.002	1.118 (0.457-2.735)	0.808	0.511 (0.161-1.626)	0.256	0.960 (0.313-2.944)	0.943
III	1.903 (1.304-2.778)	0.001	1.169 (0.460-2.968)	0.743	2.108 (0.635-6.996)	0.223	1.029 (0.343-3.082)	0.960
IV	1.071 (0.334-3.431)	0.908	1.593 (0.191-13.307)	0.667	–		–	
Unknown	2.513 (1.856-3.402)	<0.001	1.584 (0.699-3.588)	0.270	0.767 (0.328-1.793)	0.540	0.940 (0.379-2.333)	0.894
Histological subtype								
Mucinous adenocarcinoma	1		1		1		1	
Mucin-producing adenocarcinoma	0.554 (0.428-0.718)	<0.001	0.988 (0.751-1.300)	0.933	0.822 (0.488-1.385)	0.461	0.645 (0.424-0.981)	0.041
Intraductal papillary-mucinous carcinoma	0.174 (0.116-0.261)	<0.001	0.836 (0.205-3.408)	0.803	2.185 (0.288-16.606)	0.450	–	
-Others	0.294 (0.176-0.489)	<0.001	0.643 (0.203-2.036)	0.452	1.572 (0.532-4.646)	0.413	1.285 (0.306-5.398)	0.732
Tumor size								
≤3 cm	1		1		1		1	
>3 cm	1.778 (1.464-2.160)	<0.001	0.741 (0.546-1.004)	0.053	1.398 (0.890-2.198)	0.146	1.334 (0.861-2.067)	0.198
Surgery								
No	1		1		1		1	
Yes	0.209 (0.171-0.256)	<0.001	0.394 (0.162-0.958)	0.040	0.038 (0.000-6.488)	0.212	0.587 (0.081-4.231)	0.587
Radiotherapy								
No	1		1		1		1	
Yes	0.731 (0.584-0.914)	0.006	1.236 (0.633-2.414)	0.534	0.705 (0.324-1.534)	0.378	0.950 (0.555-1.627)	0.853
Chemotherapy								
No	1		1		1		1	
Yes	0.993 (0.823-1.199)	0.945	0.383 (0.292-0.503)	<0.001	0.290 (0.180-0.469)	<0.001	0.223 (0.140-0.357)	<0.001
Marital status								
Unmarried	1		1		1		1	
Married	0.822 (0.682-0.991)	0.040	0.796 (0.606-1.045)	0.796	0.831 (0.512-1.348)	0.454	0.663 (0.440-0.997)	0.048
Unknown	0.623 (0.368-1.054)	0.078	0.688 (0.399-1.184)	0.177	1.172 (0.563-2.441)	0.672	1.206 (0.431-3.375)	0.721

**Table 3:** Multivariable Cox regression analysis of cancer-specific survival in patient with IPMN with associated invasive carcinoma.

Variables	No metastasis		liver		lung		Multiple metastases	
	HR (95% CI)	P	HR (95% CI)	P	HR (95% CI)	P	HR (95% CI)	P
Age								
≤65	1		1		1		1	
>65	1.072 (0.885-1.298)	0.476	0.981 (0.728-1.322)	0.901	1.715 (1.037-2.838)	0.036	1.161 (0.688-1.5959)	0.576
Gender								
Male	1		1		1		1	
Female	0.977 (0.817-1.187)	0.817	0.962 (0.714-1.296)	0.796	1.315 (0.799-2.163)	0.281	1.485 (0.905-2.438)	0.118
Race								
White	1		1		1		1	
Black	0.859 (0.635-1.161)	0.322	0.851 (0.511-1.418)	0.535	1.126 (0.437-2.898)	0.806	1.594 (0.704-3.605)	0.263
Other	0.639 (0.462-0.884)	0.007	0.585 (0.357-0.959)	0.034	0.819 (0.219-3.060)	0.767	1.254 (0.398-3.949)	0.699
Primary site								
Head	1		1		1		1	
Body/tail	1.337 (1.077-1.659)	0.008	1.160 (0.831-1.618)	0.383	1.180 (0.675-2.063)	0.562	2.239 (1.140-4.400)	0.019
Other	1.042 (0.799-1.360)	0.760	1.197 (0.817-1.755)	0.857	1.131 (0.558-2.291)	0.733	1.638 (0.821-3.269)	0.161
Histologic grade								
I	1		1		1		1	
II	1.547 (1.101-2.173)	0.012	0.752 (0.286-1.979)	0.564	0.402 (0.101-1.595)	0.195	0.756 (0.210-2.729)	0.670
III	1.718 (1.159-2.546)	0.007	0.668 (0.232-1.923)	0.454	1.632 (0.423-6.292)	0.477	0.886 (0.244-3.220)	0.854
IV	0.782 (0.239-2.554)	0.684	3.110 (0.324-29.817)	0.325	–		–	
Unknown	1.114 (0.802-1.548)	0.520	1.055 (0.422-2.634)	0.909	0.339 (0.115-1.001)	0.050	0.933 (0.339-2.566)	0.893
Histological subtype								
Mucin-producing adenocarcinoma	1		1		1		1	
Mucinous adenocarcinoma	0.698 (0.534-0.913)	0.009	1.042 (0.775-1.402)	0.784	0.897 (0.485-1.662)	0.730	0.870 (0.549-1.381)	0.555
Intraductal papillary-mucinous carcinoma	0.350 (0.228-0.538)	<0.001	0.963 (0.216-4.295)	0.961	1.412 (0.052-38.318)	0.838	0.535 (0.112-2.564)	0.434
Others	0.556 (0.326-0.947)	0.031	0.723 (0.223-2.345)	0.590	1.839 (0.467-7.240)	0.383	–	
Tumor size								
≤3 cm	1		1		1		1	
>3 cm	1.380 (1.126-1.692)	0.002	0.803 (0.565-1.140)	0.220	1.050 (0.629-1.752)	0.853	1.593 (0.970-2.614)	0.066
Surgery								
No	1		1		1		1	
Yes	0.218 (0.171-0.278)	<0.001	0.467 (0.172-1.268)	0.135	–		1.717 (0.148-19.978)	0.666
Radiotherapy								
No	1		1		1		1	
Yes	0.663 (0.523-0.841)	0.001	1.320 (0.640-2.725)	0.452	0.767 (0.309-1.904)	0.567	1.176 (0.653-2.119)	0.589
Chemotherapy								
No	1		1		1		1	
Yes	0.770 (0.620-0.956)	0.018	0.351 (0.256-0.481)	<0.001	0.242 (0.134-0.435)	<0.001	0.191 (0.108-0.340)	<0.001
Marital status								
Unmarried	1		1		1		1	
Married	0.885 (0.725-1.080)	0.228	1.122 (0.814-1.545)	0.482	1.098 (0.620-1.944)	0.748	0.799 (0.495-1.289)	0.358
Unknown	1.077 (0.637-1.822)	0.782	0.998 (0.539-1.849)	0.995	0.997 (0.439-2.267)	0.995	2.751 (0.846-8.950)	0.093

### Risk Factor of Distant Metastases

To identify the risk factors associated with distant metastases in patients with IPMN with associated invasive carcinoma, the univariable and multivariable regression analyses were performed. In the univariable regression model, tumor primary site, histologic grade, surgery, lymph node surgery, chemotherapy, and marital status were associated with distant metastases. However, in multivariable logistic regression model after adjustment revealed that tumor located in body/tail was the independent risk factor related to distant metastases. The results were summarized in **Table 4**.

**Table 4:** Univariate and multivariate logistic regression analyses of baseline factors associated with distant metastases in patients with IPMN with associated invasive carcinoma.

Variables		Univariate analyses			Multivariate analyses		
		OR	95% CI	P	OR	95% CI	P
Age	>65 vs.≤65	1.134	0.895-1.437	0.296	0.910	0.676-1.226	0.537
Gender	Male vs. Female	1.089	0.861-1.376	0.478	0.849	0.632-1.141	0.277
Race	Black vs. White	0.791	0.532-1.176	0.247	0.662	0.411-1.066	0.089
	Other vs. White	0.693	0.468-1.027	0.068	0.783	0.485-1.264	0.317
Site	Body/tail vs. Head	3.756	2.848-4.952	<0.001	2.740	1.971-3.811	<0.001
	Other vs. Head	2.654	1.913-3.682	<0.001	1.748	1.194-2.557	0.004
Grade	II vs. I	1.520	0.858-2.692	0.151	1.175	0.583-2.367	0.653
	III vs. I	2.248	1.213-4.164	0.010	1.614	0.742-3.508	0.227
	IV vs. I	1.190	0.132-10.725	0.877	1.016	0.061-16.930	0.991
	Unknown vs. I	6.501	3.955-10.687	<0.001	1.750	0.959-3.192	0.068
Surgery	Yes vs. No	0.020	0.010-0.040	<0.001	0.055	0.021-0.142	<0.001
Lymph node surgery*	Yes vs. No	0.037	0.022-0.061	<0.001	0.445	0.204-0.971	0.042
Radiotherapy	Yes vs. No/Unknown	0.352	0.243-0.509	<0.001	0.466	0.301-0.721	0.001
Chemotherapy	Yes vs. No/Unknown	0.922	0.727-1.169	0.501	1.036	0.763-1.406	0.823
Marital status	Unmarried vs. married	0.801	0.627-1.023	0.075	0.789	0.578-1.077	0.136
	Unknown vs. married	1.914	1.103-3.323	0.021	2.335	1.095-4.981	0.028

*Regional lymph nodes have been removed by surgery.

### Construction and Validation of the Nomogram for CSS

**Figure 3:**
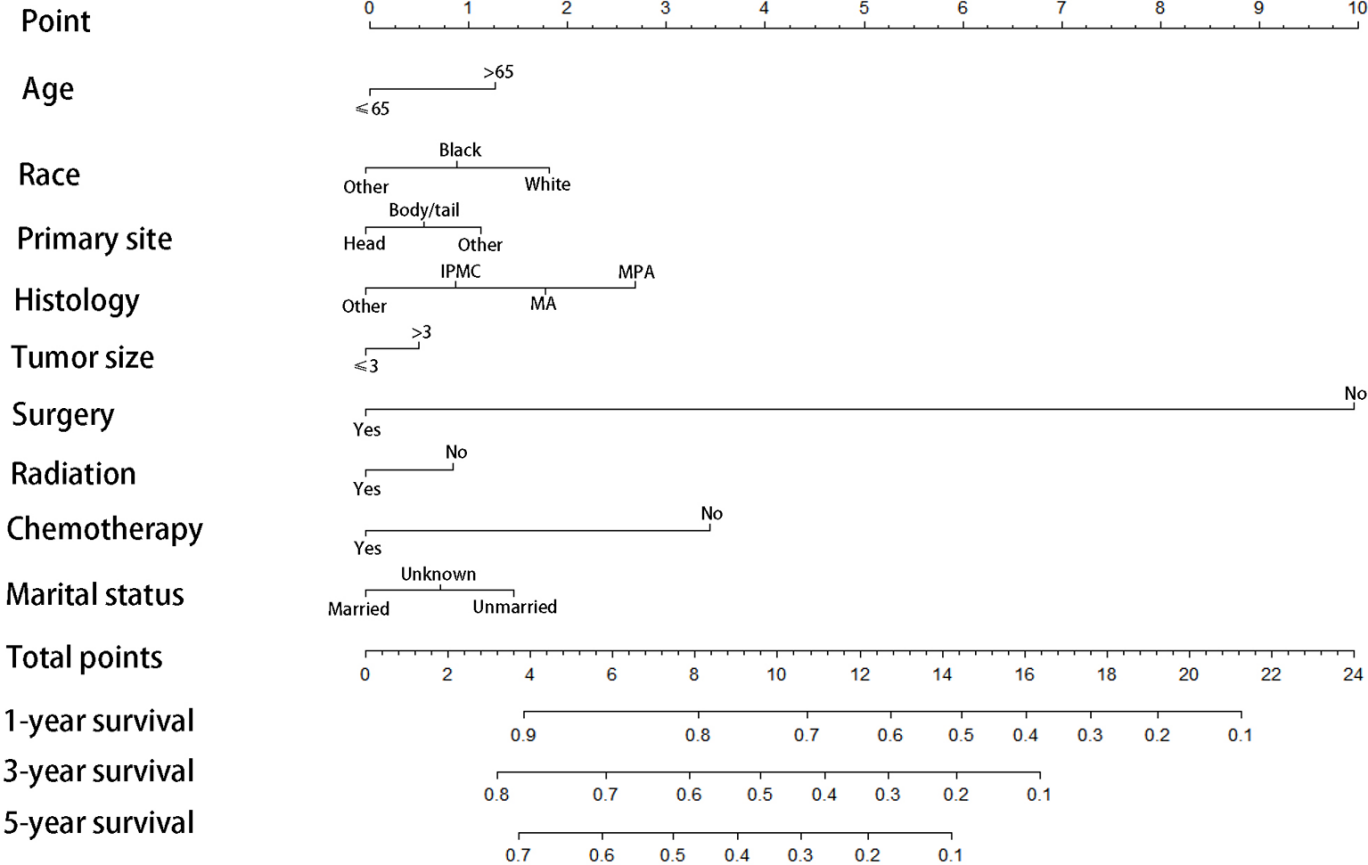
Prognostic nomogram predicting 1-, 3-, and 5-year cancer-specific survival probability for patients with IPMN with associated invasive carcinoma, IPMC, Intraductal papillary-mucinous carcinoma; MA, mucinous adenocarcinoma; MPA,mucin-producing adenocarcinoma.

**Figure 4:**
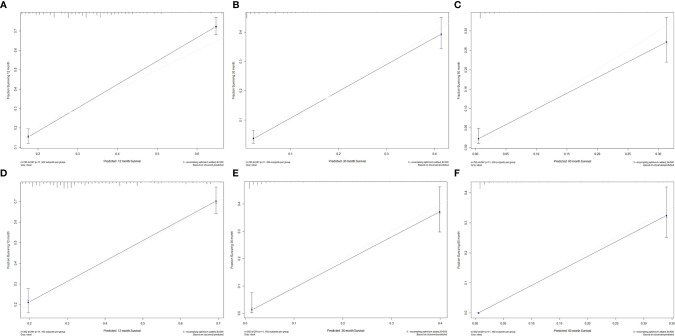
Calibration plots for 1-, 3-, and 5-year CSS in the **(A–C)** training cohort and **(D–F)** validation cohort.

We divided the whole cohort into two groups: training cohort and validation cohort. The training cohort was used to construct the nomogram for CSS and the validation cohort was used for external validation. Based on the risk factors in the multivariable Cox regression, a nomogram was constructed to predict probabilities of CSS (**Figure 3**). The C-index of nomogram in predicting CSS was 0.780 (95% CI=0.762-0.798), suggesting good discrimination of the nomogram. For the external validation of the nomogram, the C-indexes were 0.759 (95% CI=0.730–0.788) for CSS. Also, the calibration curves of CSS indicating a good agreement between predicted rates and observed probabilities (**Figure 4**).

## Discussion

IPMN with associated invasive carcinoma has a distinct poor prognosis compared with other pancreatic cyst lesions. IPMNs have been increasingly detected due to recent increased awareness and improved diagnostic modalities. To our knowledge, the current study was the first article focusing on the effect of distant metastases on the survival of patients with IPMN with associated invasive carcinoma. From our data, we concluded that around 38% of all the patients suffered distant metastases during the progression of the disease. The most common distant metastatic organ was the liver, following by multiple sites, lung, and bone. Compared with no distant metastatic subgroup, the survival time was much shorter in patients with distant metastases. Meanwhile, different prognostic factors were identified in patients with various metastatic patterns. Furthermore, tumor located in body/tail was the independent risk factor related to distant metastases.

It is widely believed that metastases were closely associated with poor outcomes in advanced malignancies. Cancer metastases include lymph node metastases and distant organ metastases. The 5-year relative survival rate of IPMN with associated invasive carcinoma was 9.7%, while that was 10% in pancreatic cancer, according to SEER Cancer Stat Facts. The 5-year relative survival rate of IPMN with associated invasive carcinoma with distant metastases was 1.7%, and the 5-year relative survival rate of pancreatic cancer was 2.9% (11). The 5-year relative survival rate of pancreatic cancer was slightly higher than that of IPMN with associated invasive carcinoma. Notably, the 5-year relative survival rate is increasing gradually the efforts in research and therapy of pancreatic cancer. In other words, the prognosis of IPMN with associated invasive carcinoma should be paid as much attention as pancreatic cancer.

Previous studies reported that lymph node metastasis is a negative factor affecting the prognosis of IPMN with associated invasive carcinoma. Schnelldorfer et al. retrospective reviewed a cohort with IPMN with associated invasive carcinoma and found that patients with lymph node metastases have a shorter median survival time of 16 months, compared with that of patients without lymph node metastases was 41 months (12). Another study performed by Maire et al. suggested that lymph node metastases were the only prognostic factor in patients with IPMN with associated invasive carcinoma after surgical resection (13). Furthermore, Partelli et al. reported that the lymph node ratio is a strong predictor of survival after resection for IPMN with associated invasive carcinoma (14). They also found that 4 patients had a recurrence in the liver. However, they did not report whether these 4 patients with IPMN had liver metastases at diagnosis. In our study, patients with isolated liver metastases are the most common in distant metastases. Some studies reported that distant metastases in patients with IPMN. Fukushima et al. reported that one of the eight patients with liver metastases at the time of first surgery (15). Yogi and colleagues reported that more than 60% of all patients with recurrent IPMN had extrapancreatic metastasis (16). Nara et al. reported that 4 patients with IPMN with associated invasive carcinoma had distant organ metastasis and 5 patients with recurrent IPMN had distant metastases (liver or lung) (17). They also compared the prognosis of IPMN and pancreatic cancer and found that there was no significant difference in the corresponding tumor-node-metastasis stages. Wang et al. reported that the median survival time of pancreatic cancer patients with liver metastases was 2 months, which is similar to the survival time of IPMN patients with liver metastases (18). The metastatic patterns of IPMN and PDAC are similar, resulting in metastasizing disproportionately to the liver (19, 20). This may be the reason why IPMN with associated invasive carcinoma has poor outcomes as pancreatic cancer. Further, we identified that tumor located in body/tail was the independent risk factor related to distant metastases. One possible explanation of our findings is that the time from diagnosis to operation was shorter for head lesions compared to body/tail lesions, increasing the probability of the body/tail lesions progressing to metastatic disease (21). This result should be validated in clinical studies with a large cohort in the future.

IPMNs of the pancreas are widely considered precursors of invasive pancreatic cancer. Based on our result and previous studies, it should be noted that the liver and lung are the most metastatic sites of IPMN with associated invasive carcinoma, which is consistent with distant metastasis of pancreatic cancer. The tendency of tumors to metastasize to specific organ sites may reflect the interaction between the underlying biology of tumor cells and the host organ microenvironment. IPMN with associated invasive carcinoma has been defined as a subtype of invasive ductal adenocarcinoma (22). IPMN with associated invasive carcinoma and pancreatic cancer have an overlapping yet distinct genetic mutation, such as KRAS and GNAS (23). Some studies attempted to unveil the molecular mechanisms of progression from early lesions to advanced malignancies. Omori et al. identified a kind of subtype that progresses to invasive pancreatic cancer *via* mutation accumulation, inheriting the KRAS and GNAS gene signature of IPMN (24). Recently, Fischer et al. revealed that early-stage IPMN contained multiple independent clones, which harbors distinct mutations. However, convergent evolution of RNF43 and TP53 mutations are acquired during later stages of tumorigenesis in IPMN (25). Inactivated RNF43 mutations are found in most IPMN (26). Interestingly, RNF43 has a negative regulatory action on the Wnt signaling, which is closely related to the underlying mechanism of pancreatic cancer with metastases (27, 28). Based on the biological mechanisms and our results, liver metastases are needed to be highlighted in the later stage of IPMN. Currently, mechanisms driving metastasis in IPMN with associated invasive carcinoma are still unclear yet. Besides, whether the liver metastasis of IPMN with associated invasive carcinoma and pancreatic cancer is a coincidence, or has the same molecular mechanism is deserved to clarify, which may better understand the malignant progress of IPMN with associated invasive carcinoma and pancreatic cancer to develop targeted drugs for distinct mutations to prolong the survival time of patients.

The therapeutic option is an essential factor influencing the prognosis of IPMN with associated invasive carcinoma. In our study, the multivariate Cox regression analysis indicated that chemotherapy could improve the survival of IPMN with associated invasive carcinoma with metastatic disease. Although surgical resection is considered the standard treatment for IPMN with associated invasive carcinoma, patients with IPMN with associated invasive carcinoma will benefit from chemotherapy. Coponi et al. retrospectively analyzed outcomes of 64 patients who received gemcitabine-based chemotherapy, concluding that patients with resected IPMN with associated invasive carcinoma might benefit from adjuvant treatment (29). Marchegiani et al. investigated the effect of adjuvant treatment on IPMN with associated invasive carcinoma and found that adjuvant therapy improved survival only in invasive carcinoma with nodal disease (30). Mcmillan et al. reported that patients with lymph node metastases got significant survival benefits from adjuvant therapy in a large series (31). More recently, a propensity analysis performed by Mungo et al. revealed that adjuvant chemotherapy was related to significantly improve overall survival in lymph node positive cases (32). Aronsson et al. performed a systematic review on the efficacy of adjuvant therapy in patients with IPMN with associated invasive carcinoma and found that adjuvant therapy is a benefit to patients based on individual tumor characteristics (33). All these results showed that a survival advantage for patients with metastatic disease when received chemotherapy. To date, there is no other study in literature that evaluated the effect of chemotherapy focusing on IPMN with associated invasive carcinoma with distant metastases.

The results of our study should be interpreted in the context of its potential limitations. First, the SEER data only provide information about bone, brain, liver, and lung; thus, we were unable to evaluate the effect of other organ metastases on the survival of patients with IPMN with associated invasive carcinoma. Therefore, there may be some patients with metastatic disease that cannot be captured in our analyses. Second, selection bias must be noted in our retrospective study, which usually occurs when the selection criteria are associated with the risk factors under investigation (34). Third, lymph node status has prominently negative on prognosis in IPMN with associated invasive carcinoma. Lymph node status for most cases was unavailable, which makes it impossible to evaluate the impact of lymph node status on prognosis in IPMN with associated invasive carcinoma. Fourth, not all cases in the SEER database were pathologically diagnosed, which may misdiagnose other pancreatic tumors such as SPN as IPMN, thereby causing confounding bias (35). Besides, the cases in SEER database cannot be classified into invasive IPMN and IPMN with concurrent PDAC. Patients with IPMN with concurrent PDAC behave as PDAC rather than invasive IPMN, which may be a potential confounding factor that may affect our analyses. In SEER database, the histologic subtypes were classified according to International Classification of Diseases for Oncology (3rd Edition). Some histologic types such as papillary carcinoma, papillary adenocarcinoma and intraductal papillary adenocarcinoma with invasion may be intraductal tubulopapillary neoplasm (ITPN) of the pancreas. In current WHO classification, ITPN was recognized as a distinct entity with tubule formation and distinct genetic aberrations (36, 37). The SEER database cannot distinguish between ITPN and IPMN, which may also be another potential confounding factor affecting our analysis results. Last, some clinical related information, such as smoking status, history of alcohol, and diabetes mellitus are not available in the SEER data, which possibly affect the results of the logistic and Cox regression analyses.

Our study first reports the information about the effect of distant metastases on patients with IPMN with associated invasive carcinoma. The liver is the most common site of distant metastases in IPMN with associated invasive carcinoma. Cox regression analyses show that tumor located in the pancreatic body/tail and chemotherapy are independent prognostic factors for CSS in patients with multiple metastases. Further, tumor located in body/tail is identified as a risk factor of distant metastases. It provides insight into the similar distant metastatic pattern between IPMN with associated invasive carcinoma and pancreatic cancer. Due to the limitations mentioned above, further trials are needed to investigate the metastatic pattern in IPMN with associated invasive carcinoma.

## Data Availability Statement

The original contributions presented in the study are included in the article/supplementary material. Further inquiries can be directed to the corresponding author.

## Author Contributions

XH wrote the manuscript. XH and SY participated in data collection, manuscript drafting, table/figure creation, and manuscript revision. GD, XL and JW assisted with performing the experiments. YG helped to analyze the data. JZ designed the study and supervised the work. XH and SY contributed with essential ideas and discussion. All authors contributed to the article and approved the submitted version.

## Conflict of Interest

The authors declare that the research was conducted in the absence of any commercial or financial relationships that could be construed as a potential conflict of interest.
